# Brain dysfunction as one cause of CFS symptoms including difficulty with attention and concentration

**DOI:** 10.3389/fphys.2013.00109

**Published:** 2013-05-20

**Authors:** Benjamin H. Natelson

**Affiliations:** ^1^Director, Pain and Fatigue Study Center, Department of Pain Medicine and Palliative Care, Beth Israel Medical Center, ManhattanNew York, NY, USA; ^2^Professor of Neurology, Albert Einstein College of Medicine, BronxNew York, NY, USA

**Keywords:** fatigue, pathophysiology, oxidative stress, causative hypotheses, syndrome

## Abstract

We have been able to reduce substantially patient pool heterogeneity by identifying phenotypic markers that allow the researcher to stratify chronic fatigue syndrome (CFS) patients into subgroups. To date, we have shown that stratifying based on the presence or absence of comorbid psychiatric diagnosis leads to a group with evidence of neurological dysfunction across a number of spheres. We have also found that stratifying based on the presence or absence of comorbid fibromyalgia leads to information that would not have been found on analyzing the entire, unstratified patient group. Objective evidence of orthostatic intolerance (OI) may be another important variable for stratification and may define a group with episodic cerebral hypoxia leading to symptoms. We hope that this review will encourage other researchers to collect data on discrete phenotypes in CFS to allow this work to continue more broadly. Finding subgroups of CFS suggests different underlying pathophysiological processes responsible for the symptoms seen. Understanding those processes is the first step toward developing discrete treatments for each.

Chronic fatigue syndrome (CFS) is a medically unexplained illness characterized by new onset of fatigue, severe enough to produce a substantial decrease in activity, and accompanied by infectious, rheumatological, and neuropsychiatric symptoms (Fukuda et al., 1994). One of the most common and disabling symptoms is the subjective complaint of “brain fog” or inability to concentrate and do multiple tasks at the same time. Our earliest study provided objective evidence of a problem with attention and concentration (DeLuca et al., 1993).

Because CFS is defined using a clinical case definition, patients with this diagnosis must constitute a heterogeneous group, probably with multiple causative agents—much as was the case of the eighteenth century diagnosis of dropsy. The condition of dropsy evolved to a host of more specific diagnoses including congestive heart failure and other organ failures when the pathological causes of the specific organ dysfunction leading to dropsy were discovered. Our approach has been to try to stratify along certain phenotypic criteria to try to develop more homogeneous subgroups. In contrast, some researchers have been impressed by the large amount of comorbidity occurring among several of these syndromes—namely, CFS and fibromyalgia (FM). Confirming this with data from our Center, we found ~37% of patients with CFS also fulfilled case criteria for FM (Ciccone and Natelson, 2003), and one study of patients with FM reported that about 20% also fulfilled criteria for CFS (Buchwald and Garrity, 1994). Regarding this overlap between syndromes, Wessely et al. (1999), for example, have suggested that the “similarities between them outweigh the differences” and Barsky and Borus (1999), noting similarities between CFS and FM, suggested an explanatory model based on the concept of symptom amplification—that is, a common psychological tendency to somatize or misconstrue the significance of normal physical sensation. Moving from the question as to whether CFS and FM are the same or different illnesses is the conclusion that many patients with CFS have their illness based on somatization. We did explore this hypothesis early on and learned that the probability of any one patient receiving the diagnosis of Somatization Disorder depended on whether or not his/her symptoms were coded as psychiatric or physical. If the coder decided the infectious, rheumatological, and neuropsychiatric symptoms of CFS were psychiatric, then nearly every patient would be classified as having Somatization Disorder, but if the symptoms were coded as physical, then nearly none would be so classified. Based on this assessment, we eschewed the idea of “lumping” patients into one explanatory rubric and have continued our efforts to “split” patients into discrete subgroups.

Over the years, we have used two major strategies for stratifying patients. The first used the presence or absence of comorbid psychiatric diagnosis—usually major depressive disorder. Our first target was the neuropsychological dysfunction we had originally reported (DeLuca et al., 1993). In a follow up study, we stratified our patients into those with or without comorbid Axis I diagnoses: we found that the group with no comorbid psychiatric diagnoses was the group with the worst neuropsychological test results (DeLuca et al., 1997). After doing an initial study which showed increased numbers of anatomic abnormalities on brain magnetic resonance imaging (Natelson et al., 1993), we followed that study up after stratifying our patients into PSYCH or NO-PSYCH groups. Again, we found that the patients without psychiatric comorbidity were the ones with the highest number of abnormalities—usually small, circumscribed areas of T2 signal in frontal lobes (Lange et al., 1999). To start determining whether these were insignificant epiphenomena or perhaps functioned to affect physical function, we asked if physical function as assessed by the SF-36 correlated with MRI findings and in fact found that those with the lesions had worse physical function than those with normal brain MRIs (Cook et al., 2001). Our findings of increased numbers of abnormalities in the NO-PSYCH patients are of interest when compared to a subset analysis of an earlier study (Greco et al., 1997): while no significant difference in rate of abnormalities was found between a group of unstratifed patients and controls, they did report a difference significant at the 0.06 level between a subgroup of patients without depression compared to healthy controls; thus our data do replicate this finding. The sum of these data suggests that some CFS patients may have a brain marker of their disease in the form of small nonspecific MRI white matter abnormalities, occurring predominantly in the frontal lobes. These studies led to our current working hypothesis—that a subgroup of patients with CFS has an underlying neurological disease which leads to the symptoms of fatigue and cognitive dysfunction.

Using the same stratification strategy, we moved on to other studies. First, we collected spinal fluid from 45 CFS patients and 13 healthy controls. As expected, the healthy controls had white blood counts per high power field and protein concentrations that were all below the high value for laboratory normals. In contrast, we found that nearly 30% of the patients had elevations in one or both of these variables. While there was no difference in lifetime rates of Axis I pathology, the group with the abnormal spinal fluids had significantly fewer cases with a diagnosis of current depression than the group with the normal spinal fluids (Natelson et al., 2005). Our next study looked at cerebral blood flow in CFS (Yoshiuchi et al., 2006). We found that CFS patients as a group had significant reductions in cerebral blood flow across a number of brain in regions. However, those patients with no comorbid psychiatric diagnosis had more areas of reduced blood flow compared to controls than patients who did have comorbid psychiatric diagnoses. Finally, we re-evaluated data on ventricular lactate which had shown increases for CFS relative to controls (Mathew et al., 2009). Nine of the 16 CFS patients fell into a “high” group (i.e., displaying higher ventricular lactate levels that were one SD above the CFS group mean) which was higher than every healthy volunteer and was responsible for the overall group effect. In exploratory *post-hoc* analyses, these 9 CFS patients were compared with the remaining 6 CFS patients with normal lactate levels (“normal”), which revealed that the only differences between the two CFS subgroups was greater overall functional disability on the SF-36 (*P* = 0.039), and a trend toward greater lifetime Axis I mood disorder comorbidity (4 of 7 vs. 1 of 9, Fisher Exact probability test, *P* = 0.07) in the CFS group with “low” lactate levels compared to CFS patients with higher ventricular lactate (Mathew et al., unpublished data). Although preliminary, these data suggest that “no-psych” patients may have an underlying encephalopathy producing the symptoms of CFS.

The other way we have stratified our CFS patient sample is on the presence or absence of fibromyalgia—characterized by the patient reporting widespread pain and having tenderness in at least 11 of 18 tender points subjected to palpation (Wolfe et al., 1990). When comparing CFS patients with and without comorbid fibromyalgia, we found that only patients in the CFS only group and not those with both CFS plus fibromyalgia had abnormal neuropsychological test results (Cook et al., 2005). A recent paper from another group corroborated our finding of normal cognitive function in fibromyalgia (Mohs et al., 2012). Unfortunately, we do not have data as to psychiatric status for these subjects; however other studies have revealed that patients with CFS only have approximately half the psychiatric comorbidity than those with comorbid FM (Ciccone and Natelson, 2003); these data support our working hypothesis that the group of CFS patients with no co-existing psychiatric problems has an underlying brain disease.

These results support our hypothesis that stratifying CFS patients into subgroups can lead to improved understanding of the cognitive complaints of all CFS patients. We have two major hypotheses for how the changes in blood flow affect neural functioning adversely, leading to increased ventricular lactate and/or small lesions in frontal white matter, and then to symptoms. The first of these, spearheaded by my colleague Dr. Dikoma Shungu of Weil-Cornell, is an outgrowth of Martin Pall's hypothesis (Pall, 2000) that some immunological or infectious trigger activates one or more pro-inflammatory cytokines which induce a synthesis chain leading to the production of peroxynitrite, a potent pro-apoptotic, and pro-inflammatory reactive oxygen or nitrogen species (Hoffman et al., 1997; Basu, 2008). This process is inhibited by adequate antioxidant capacity/reserves, of which glutathione (GSH) is the most abundant and important component. In the face of inadequate levels of antioxidants, peroxynitrite will react with arachidonic acid in cell membrane phospholipids, to form isoprostanes, compromising membrane and cell function. A final metabolic step frees the isoprostanes from the cell membrane phospholipids, allowing them to move into body fluids, where they can be measured to provide an objective and reliable measure for the presence of oxidative stress.

This model is pertinent to CFS for the following reasons: first, two studies have reported that isoprostanes are elevated in CFS (Kennedy et al., 2005; Robinson et al., 2009). And second, we have found that cortical GSH is reduced by 36% in CFS (Shungu et al., 2012). These data lead to the inference that brain oxidative stress molecules are increased in CFS; these molecules have potent vasoconstrictor effects on cerebral arterioles (Hoffman et al., 1997) and thus could explain the decreases in regional cerebral blood flow that we have now found in a number of studies (Yoshiuchi et al., 2006; Biswal et al., 2011; Shungu et al., 2012). Finally the impaired cellular metabolism produced by these molecules could, via co-occurring increases in anaerobic glycolytic activity, lead to the increased ventricular CSF lactate in CFS shown to occur by Shungu and colleagues in three separate studies (Mathew et al., 2009; Murrough et al., 2010; Shungu et al., 2012). One possibility that our studies have ruled out is mitochondrial dysfunction from the accumulation of free radicals: we found brain ATP and associated high energy phosphates to be normal in CFS (Shungu et al., 2012). Thus, we are focusing on the oxidative stress hypothesis in this model of CFS.

The second explanatory hypothesis is directed to the ~25% of CFS patients with physiological evidence of orthostatic intolerance (OI). Early work by David Streeten (Streeten and Anderson, 1992) tied chronic symptoms including fatigue to a form of OI characterized by delayed hypotension induced by orthostatic stress. Subsequent work made the link with CFS directly. The researchers did tilt testing with pharmacological potentiation in young adults with CFS and reported that most patients developed delayed hypotension accompanied by increased fatigue compared to healthy controls (Bou-Holaigah et al., 1995). While several groups have replicated this finding in older patient populations (DeLorenzo et al., 1997; Freeman and Komaroff, 1997; Schondorf et al., 1999), even more groups have not, including the original research team reporting the finding (Razumovsky et al., 2003), another group studying twins (Poole et al., 2000), yet another studying a community sample of CFS patients (Jones et al., 2005), and our own carefully controlled study of unmedicated and uninstrumented patients (LaManca et al., 1999).

Subsequently attention has turned away from delayed hypotension to orthostatic tachycardia—defined as an increase in heart rate from those in the supine position of ≥30 beats per minute or rates of ≥130 beats per minute while standing. Later studies suggested an important age factor in determining the physiological form of OI. Almost every adolescent with CFS had orthostatic tachycardia (Stewart et al., 1999), but this was not the case with adults (Natelson et al., 2007) where rates appear to be lower ranging from 10% (Naschitz et al., 2006) to 27% (Hoad et al., 2008) with the higher rate found in UK patients who reported having the diagnosis of CFS rather than being directly diagnosed. In our own study of adult patients (Natelson et al., 2007), orthostatic tachycardia was not common, occurring in only 11% of patients, not significantly different from rates in healthy controls.

While orthostatic tachycardia is not common in adults, orthostatic hyperventilation—end tidal CO_2_ values lowered to below 30 mmHg during orthostatic challenge—seems to be substantially more common in that we found it occurred in 21% of adult CFS patients compared to 3% in healthy controls (Natelson et al., 2007). When we broke out the data by gender, we found orthostatic hyperventilation to occur in 35% of the female patients—a very substantial proportion (Natelson et al., 2007). The patients with orthostatic hyperventilation showed a progressive fall in end tidal CO_2_ during orthostatic challenge—a trend also seen, albeit much milder, in CFS patients without OI or in healthy controls. In a parallel study, a group of Israeli investigators (Naschitz et al., 2006) also monitored end tidal CO_2_ in CFS patients and found a rate of 31% showing hypocapnia (gender not specified).

The common explanation for hyperventilation is anxiety. We specifically asked volunteers in our study to rate their anxiety on a visual analog scale (Natelson et al., 2007). While patients both with and without OI had higher anxiety levels while supine than controls that difference did not increase in the OI group during orthostatic challenge. In fact, there were no differences in groups apart from the initial shift noted at baseline. Based on that result, we hypothesized that orthostatically-induced hyperventilation represented a physiological response to intra-thoracic hypovolemia related to blood pooling distally. Novak et al. have come to a similar conclusion in their studies of cerebral blood flow during orthostatic challenge in syncope patients. They postulated that patients hyperventilate for several reasons: first, to help pull blood into the chest by increasing pre-load mechanically and next to combat decreases in blood pressure by tachypnea-induced vasoconstriction (Novak et al., 1998).

Finding orthostatic tachycardia or hyperventilation is a physiological confirmation of the complaint of OI. While we do not know whether physiological manifestation of OI correlates with plasma isoprostanes or, inversely, with brain GSH (we are currently doing studies to answer this question), another possibility exists. Using two methods for determining absolute cerebral blood flow, we have demonstrated reductions in CFS patients as a group in the resting state (Yoshiuchi et al., 2006; Biswal et al., 2011). We reported average blood flow and thus some patients had blood flow in the normal range while others had flow that was quite reduced relative to controls. We hypothesize that the OI group may be the ones with the reduced brain blood flow at rest and that this group of patients may show further decreases in brain blood flow during orthostatic challenge. Only one study in CFS patients exists to date on the effects of orthostatic challenge on some measure of brain blood flow—i.e., middle cerebral arterial blood velocity assessed by transcranial Doppler (Razumovsky et al., 2003). Using this technique, no significant difference was found in resting middle cerebral artery blood velocity between patients and controls in the supine posture, and no difference was found during orthostatic stress. However, only 10% of patients in this study had OI, and this rate was the same as that in controls. Not finding differences in either supine or challenged postures may have two explanations: first, cerebral blood velocity may not reflect the same measure as absolute cerebral blood flow and second, it may be important to have a larger group of patients with OI and then to compare blood flow in that group to those without OI. If that experiment were done, we might expect that the patients with OI but not those without OI will have dramatic decreases in cerebral blood flow with commensurate increases in resistance due to orthostatically-induced hyperventilation.

If this is the case, then we would expect that it is the patients with OI who are at increased risk of developing the increased T2-signal lesions we have reported to occur via a process leading to sporadic cerebral hypoxia. As a corollary, we would expect that it is these patients who have the most difficulty with cognitive processing. Unfortunately, we had not been assessing CFS patients for OI at the time of these earlier studies—a deficiency we are now remedying in current work. We expect that OI will be more common in patients with no history of psychiatric diagnosis than in those with such diagnoses.

In conclusion, we have been able to reduce substantially patient pool heterogeneity by identifying phenotypic markers that allow the researcher to stratify patients into subgroups. To date, we have shown that stratifying based on the presence or absence of comorbid psychiatric diagnosis leads to a group with evidence of neurological dysfunction across a number of spheres. We have also found that stratifying based on the presence or absence of comorbid fibromyalgia leads to information that would not have been found on analyzing the entire, unstratified patient group. Objective evidence of OI may be another important variable for stratification and may produce some of the symptoms of CFS via intermittent cerebral hypoxia. We hope that this review will encourage other researchers to collect data on discrete phenotypes in CFS to allow this work to continue more broadly. Finding subgroups of CFS suggests different underlying pathophysiological processes responsible for the symptoms seen. Understanding those processes is the first step toward developing discrete treatments for each.

## Conflict of interest statement

The author declares that the research was conducted in the absence of any commercial or financial relationships that could be construed as a potential conflict of interest.
